# Role of Protein Glycosylation in *Candida parapsilosis* Cell Wall Integrity and Host Interaction

**DOI:** 10.3389/fmicb.2016.00306

**Published:** 2016-03-08

**Authors:** Luis A. Pérez-García, Katalin Csonka, Arturo Flores-Carreón, Eine Estrada-Mata, Erika Mellado-Mojica, Tibor Németh, Luz A. López-Ramírez, Renata Toth, Mercedes G. López, Csaba Vizler, Annamaria Marton, Adél Tóth, Joshua D. Nosanchuk, Attila Gácser, Héctor M. Mora-Montes

**Affiliations:** ^1^Departamento de Biología, División de Ciencias Naturales y Exactas, Universidad de GuanajuatoGuanajuato, Mexico; ^2^Department of Microbiology, University of SzegedSzeged, Hungary; ^3^Centro de Investigación y de Estudios Avanzados del Instituto Politécnico NacionalIrapuato, Mexico; ^4^Biological Research Centre, Hungarian Academy of SciencesSzeged, Hungary; ^5^Department of Medicine and Department of Microbiology and Immunology, Albert Einstein College of MedicineBronx, NY, USA

**Keywords:** cell wall, mannosylation pathway, *Candida parapsilosis*, host-fungus interplay, virulence, mannosyltransferase

## Abstract

*Candida parapsilosis* is an important, emerging opportunistic fungal pathogen. Highly mannosylated fungal cell wall proteins are initial contact points with host immune systems. In *Candida albicans*, Och1 is a Golgi α1,6-mannosyltransferase that plays a key role in the elaboration of the *N*-linked mannan outer chain. Here, we disrupted *C. parapsilosis OCH1* to gain insights into the contribution of *N-*linked mannosylation to cell fitness and to interactions with immune cells. Loss of Och1 in *C. parapsilosis* resulted in cellular aggregation, failure of morphogenesis, enhanced susceptibility to cell wall perturbing agents and defects in wall composition. We removed the cell wall *O*-linked mannans by β-elimination, and assessed the relevance of mannans during interaction with human monocytes. Results indicated that *O-*linked mannans are important for IL-1β stimulation in a dectin-1 and TLR4-dependent pathway; whereas both, *N-* and *O-*linked mannans are equally important ligands for TNFα and IL-6 stimulation, but neither is involved in IL-10 production. Furthermore, mice infected with *C. parapsilosis och1*Δ null mutant cells had significantly lower fungal burdens compared to wild-type (WT)-challenged counterparts. Therefore, our data are the first to demonstrate that *C. parapsilosis N*- and *O*-linked mannans have different roles in host interactions than those reported for *C. albicans*.

## Introduction

*Candida parapsilosis* is an opportunistic fungal pathogen largely associated with nosocomial infections in newborns and immunocompromised patients (Nosek et al., 2009). Over the past two decades, the incidence of *C. parapsilosis* has dramatically increased, such that this organism is the second most commonly isolated *Candida* species from blood cultures; though it is considered less virulent than *C. albicans* (Trofa et al., 2008). Moreover, increased resistance to some antifungal drugs, such as echinocandins, occurs in this pathogen (Trofa et al., 2008). In order to develop new and more effective drugs, important efforts are currently underway to achieve a better understanding of how fungal invasion occurs, including the pathogenesis of *C. parapsilosis*, as well as to identify new antifungal targets.

The cell wall is the first site of interaction between most fungal pathogens and host cells, so this structure profoundly influences the recognition of fungal cells and can trigger a protective immune defense (Netea et al., 2008; Díaz-Jiménez et al., 2012). At present, little is known about the organization and composition of the *C. parapsilosis* cell wall; nevertheless, as this fungus is closely related to *C. albicans*, it has been assumed that this organelle is similar in both organisms. In *C. albicans*, the cell wall is composed of an inner layer of chitin and β1,3- and β1,6-glucans that is covered with an outer layer of highly glycosylated proteins (Díaz-Jiménez et al., 2012). These proteins are covalently modified with mannose-rich oligosaccharides attached to either asparagine (*N*-linked mannans) or serine/threonine (*O*-linked mannans) residues (Klis et al., 2001). Mannans play important roles in cell wall integrity, adhesion to host tissues, virulence and in the establishment of a protective host immune response (Bates et al., 2005, 2006, 2013; Mora-Montes et al., 2007, 2009, 2010; Díaz-Jiménez et al., 2012; Hall et al., 2013; West et al., 2013). The interaction of *C. albicans* with immune cells has been thoroughly studied to establish the mechanisms underlying the generation of an effective anti-*Candida* immune response. Although, less well studied in *C. parapsilosis*, certain key differences in the phagocytic process and induction of the T-cell response have already been identified (Toth et al., 2013, 2014).

Thus, far, it is well established that in *C. albicans* the β1,3-glucans, *N-* and *O*-linked mannans are the main pathogen-associated molecular patterns (PAMPs) recognized by the immune system (Brown and Gordon, 2001; Netea et al., 2006). The mannose receptor (MR), expressed at the surface of dendritic cells, monocytes, and macrophages, recognizes α-mannose residues within the *N*-linked mannans (Netea et al., 2006; McKenzie et al., 2010). Other immune receptors for *N*-linked mannans include DC-SIGN (Cambi et al., 2008), Mincle (Wells et al., 2008), dectin-2 (Saijo et al., 2010), and dectin-3 (Zhu et al., 2013). In addition, the phosphomannan moiety contained in both *N*- and *O*-linked mannans is a key cell wall component for the effect of antimicrobial peptides, and for proper phagocytosis by murine macrophages (Harris et al., 2009; McKenzie et al., 2010). The *O*-linked mannans are recognized by the toll-like receptor 4 (TLR4), while β1,3-glucan is sensed through dectin-1 and TLR2 (Brown and Gordon, 2001; Netea et al., 2006).

Mannan relevance for *C. albicans* cell wall integrity, virulence, and sensing by innate immune cells has been mainly assessed using mutant cells lacking specific enzymes with key roles during the assembly of either *N*- or *O*-linked mannans (Bates et al., 2005, 2006, 2013; Munro et al., 2005; Prill et al., 2005; Mora-Montes et al., 2007, 2010; Hall et al., 2013). Among them, disruption of the *OCH1* gene has been a valuable molecular tool to understand those cellular processes (Bates et al., 2006). This gene encodes for a Golgi-resident α1,6-mannosyltransferase that initiates the elaboration of the *N-*linked mannan outer chain. Null mutants lacking this gene in *C. albicans* exhibit reduced virulence, increased sensitivity to cell-wall perturbing agents, and reduced ability to stimulate cytokine production by human mononuclear cells (PMBCs) and dendritic cells (Bates et al., 2006; Netea et al., 2006; Cambi et al., 2008). Moreover, cell treatments with endoglycosidase H (endo H; an enzyme that trims the *N*-linked glycans from glycoproteins, Kobata 1979) or β-elimination, which specifically removes *O*-linked mannans, have further contributed to the understanding of the physiological role of these cell wall components in the biology of *C. albicans* (Hamada et al., 1981; Hazen and Glee, 1994; Mormeneo et al., 1994; Goins and Cutler, 2000; Spreghini et al., 2003).

In contrast, the role of *C. parapsilosis* mannans in cell fitness, virulence and immune sensing is unknown. Here, we disrupted *C. parapsilosis OCH1* and found that loss of proper *N*-linked mannosylation affected cell morphology, filamentation, wall composition, susceptibility to wall perturbing agents, and fungal interaction with human PBMCs. Furthermore, we demonstrated that *O*-linked mannans are not required for phagocytosis by human monocyte-derived macrophages, but they play a central role in the stimulation of IL-1β by human PBMCs. Notably, the production of this cytokine required the co-stimulation of dectin-1 and TLR4. In a murine systemic infection model, but not in the alternative invertebrate model *Galleria mellonella*, the *C. parapsilosis och1*Δ null mutant showed significant defects in virulence.

## Results

### Deletion of *CpOCH1*

**Figure 1:**
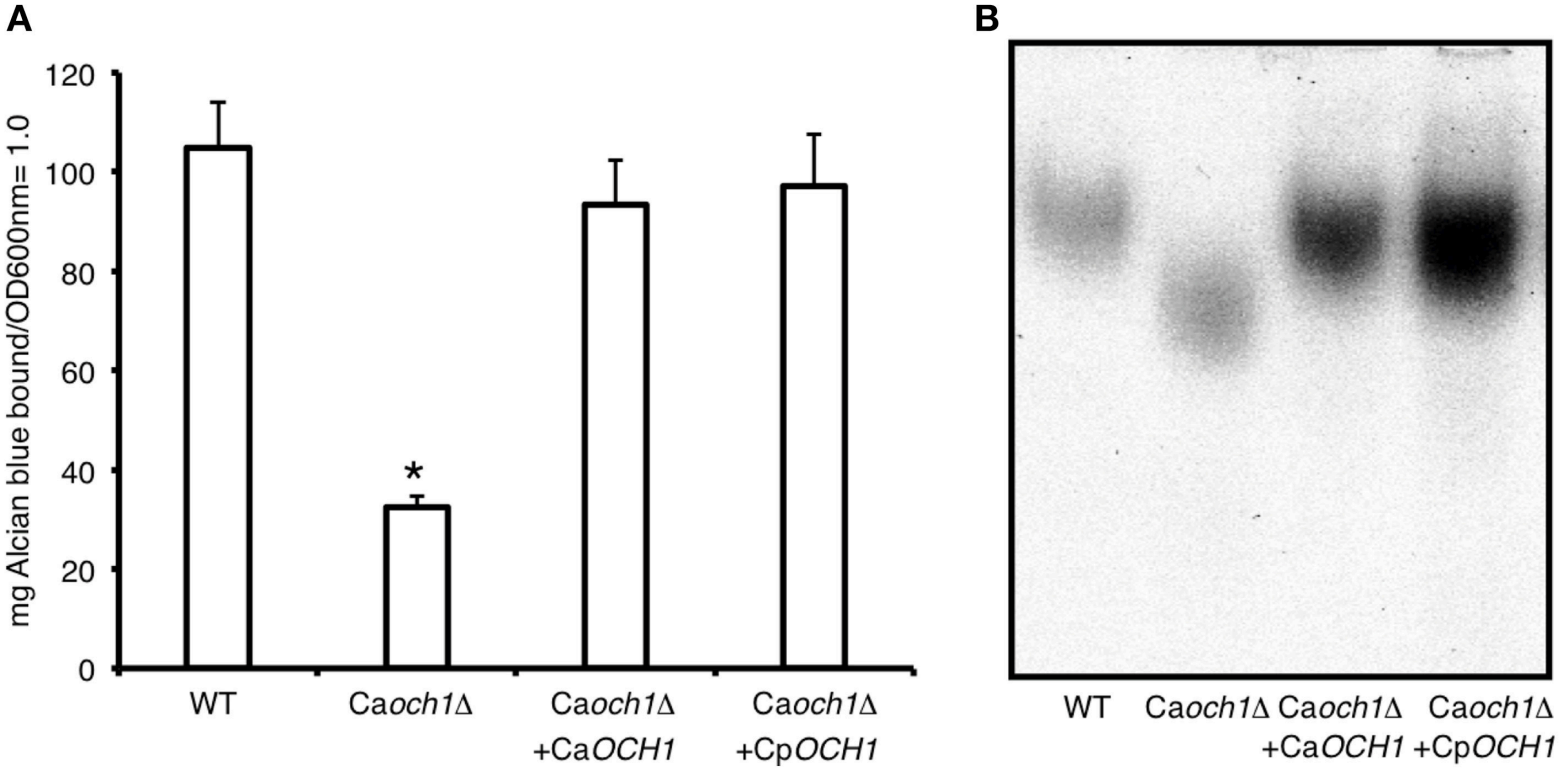
**Cp*OCH1* is the functional ortholog of Ca*OCH1***. The Cp*OCh1* open reading frame was expressed in the Ca*och1*Δ null mutant, as described in materials and methods, and the ability of cells to bind Alcian blue, an indirect measurement of the cell wall phosphomannan content, and therefore of mannan length was analyzed **(A)**. In addition, cells were grown in minimal medium with GlcNAc added to induce the *N*-acetylhexosaminidase activity, broken to obtain a cell homogenate and protein samples were used to perform electrophoretic mobility shift assays of Hex1, a highly *N*-linked glycosylated *N*-acetylhexosaminidase **(B)**. ^*^P < 0.05. Strains used are: NGY152 (WT), Ca*och1*Δ (NGY357); Ca*och1*Δ + Ca*OCH1* (NGY328), Ca*och1*Δ + Cp*OCH1* (HMY163).

Cp*OCH1* was identified in the *Candida* genome database (http://www.candidagenome.org/) by homology to the *C. albicans* ortholog (Systematic name orf19.7391). The Cp*OCH1* open reading frame of 1089 bp (Systematic name CPAR2_404930) is predicted to encode a type-II transmembrane protein of 362 amino acids of the glycosyl transferase family 32, which shows 67 and 78% of identity and similarity to *C. albicans* Och1, respectively. This open reading frame is unlikely to encode the closely related α1,6-mannosyltransferase Hoc1, as it shows 39 and 57% of identity and similarity to *C. albicans* Hoc1 (Systematic name orf19.3445). The Cp*OCH1* alleles were deleted by sequential gene replacement in the CPL2H1 strain (Holland et al., 2014) as described for *C. albicans*, using disruption cassettes complementing leucine and histidine auxotrophies (Figure 1SA; Noble et al., 2010). Southern blotting assays were performed to discard ectopic integrations within the *C. parapsilosis* genome, using specific probes for Cm*LEU2*, Cd*HIS1* and Cp*OCH1*. Results showed loss of *CpOCH1* gene and a single copy of each replacing cassette within the mutant strain genome (Figure 1SB), demonstrating the production of a *C. parapsilosis och1*Δ null mutant. As a control, the Cp*OCH1* open reading frame, under the control of the *C. albicans TDH3* promoter was re-integrated into the *C. parapsilosis och1*Δ null mutant, generating a re-integrant control strain. In order to demonstrate that Cp*OCH1* is the functional ortholog of the Ca*OCH1*, we complemented a *C. albicans och1*Δ null mutant (Bates et al., 2006) with Cp*OCH1*. Results indicated that the *C. parapsilosis* gene was able to restore the levels of phosphomannosylation (Figure 1A) and the electrophoretic mobility of Hex1 (Figure 1B), a secreted protein previously used to assess the status of the *N*-linked glycosylation pathway (Bates et al., 2005, 2006; Mora-Montes et al., 2007; Lopes-Bezerra et al., 2015). Therefore, *C. parapsilosis OCH1* is the functional ortholog of *C. albicans OCH1*.

### Filamentation, colony and cell morphology of the *C. parapsilosis och1*Δ null mutant

**Figure 2:**
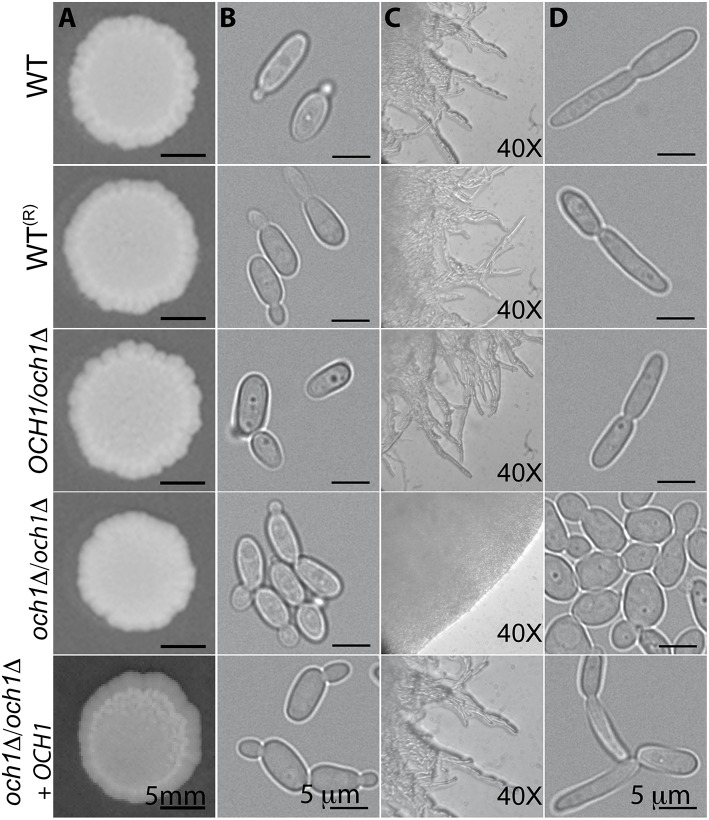
**Loss of *OCH1* affects *C. parapsilosis* morphological transition**. Cells from the Cp*och1*Δ null mutant grown on dextrose-Sabouraud plates at 28°C did not show differences in colonial morphology **(A)**, but tended to form aggregates in dextrose-Sabouraud broth **(B)**. Upon growth in RPMI 1640 plates supplemented with 10% fetal bovine serum at 37°C, the null mutant displayed rounded and well-defined colony edges, whereas the control strains generated filamentous projections outside the colony **(C)**. When filamentation was induced using liquid medium (RPMI 1640 added with 10% fetal bovine serum and 37°C), control strains underwent morphological transition, but Cp*och1*Δ null mutant was unable to form pseudohyphae **(D)**. The strains used are: CLIB-214 (WT), CPRI (WT^(*R*)^), AP (*OCH1*/*och1*Δ), AP-1 (*och1*Δ*/och1*Δ), and AP-2 (*och1*Δ*/och1*Δ + *OCH1*).

The growth rate of the Cp*och1*Δ null mutant was significantly reduced, with doubling times of 2.79 ± 0.22 h for the null mutant vs. 1.54 ± 0.11 and 1.57 ± 0.15 h for the wild-type (WT) and reintegrant control strains, respectively (*P* < 0.05). Experiments conducted in presence of 2 units/mL chitinase to disrupt cell aggregates (Bates et al., 2006) showed similar results (data not shown). The Cp*och1*Δ null cells displayed a clumpy phenotype, i.e., they tended to form cell aggregates when cultured in liquid media (Figure 2B). Colony morphology was not significantly affected when cells were grown on Sabouraud plates at 20 and 28°C, or under pH conditions ranging from 4 to 8 (Figure 2A and data not shown). However, when strains were grown at 37°C, the null mutant failed to develop pseudohyphae, even when cultured in filamentation-inducing media, such as RPMI supplemented with 10% (v/v) fetal bovine serum, Lee or Spider media (Figure 2D, and data not shown). Under such growth conditions, the Cp*och1*Δ null mutant displayed well-defined colony edges on the plates and no filamentous structures were visible by light microscopy (Figure 2C). The reintegrant control strain did not show the clumpy phenotype and effectively generated pseudohyphae, confirming that the phenotypes observed in the null mutant strain are indeed related to *OCH1* disruption. Therefore, loss of *OCH1* affects *C. parapsilosis* morphogenesis.

### Loss of CpOch1 affects cell wall composition and susceptibility to cell wall perturbing agents

**Figure 3:**
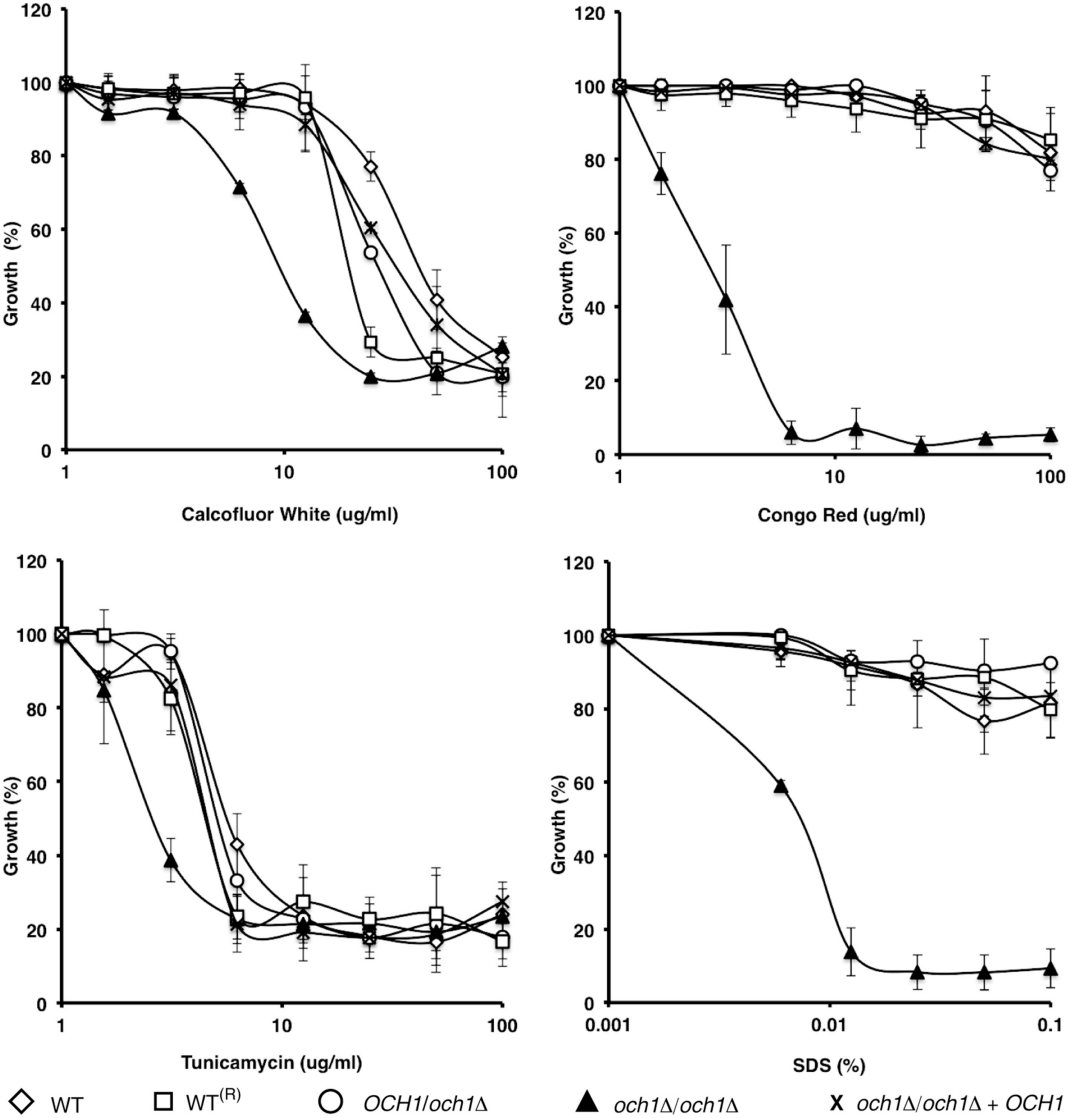
***C. parapsilosis och1*Δ null mutant displays enhanced susceptibility to specific cell wall perturbing agents**. The wild type (open diamonds), wild-type plus Cm*LEU2* and Cd*HIS1* (open squares), *OCH1*/*och1*Δ (open circles), *och1*Δ/*och1*Δ (closed triangles), and *och1*Δ + *OCH1* (X symbol) cells were incubated, using a micro-dilution method, with different concentrations of either Calcofluor White, Congo Red, Tunicamycin, or SDS, and growth was determined after incubation for 16 h at 30°C. Growth data were normalized as percentage of those generated with the same strains without treatment. The Cp*och1*Δ null mutant (closed triangles) exhibited a higher susceptibility to all the perturbing agents tested. Data are means ± SD of three independent experiments performed in duplicates.

To assess the effect of Cp*OCH1* mutation on cell wall integrity, we tested the susceptibility of the null mutant to a range of cell wall perturbing agents and compounds associated with glycosylation defects. The Cp*och1*Δ null mutant exhibited enhanced susceptibility to Calcofluor White and Congo Red (*P* = 0.04055 and 0.00124, respectively), which interact with cell wall chitin and β-glucans, respectively (Figure 3). Furthermore, the null mutant had an increase in susceptibility to Tunicamycin (*P* = 0.0278), an inhibitor of the first steps during *N*-linked mannan biosynthesis; and it was highly susceptible to SDS (*P* = 0.0074), a detergent that affects the plasma membrane (Bates et al., 2006; Mora-Montes et al., 2007); whereas the WT and control strains were largely resistant (Figure 3). Hygromycin B, vanadate, and osmotic stressors such as NaCl and KCl were also tested, but no significant differences were observed (data not shown). Similar results were generated when the experiments were performed in presence of chitinase to disaggregate cells (not shown).

**Figure 4:**
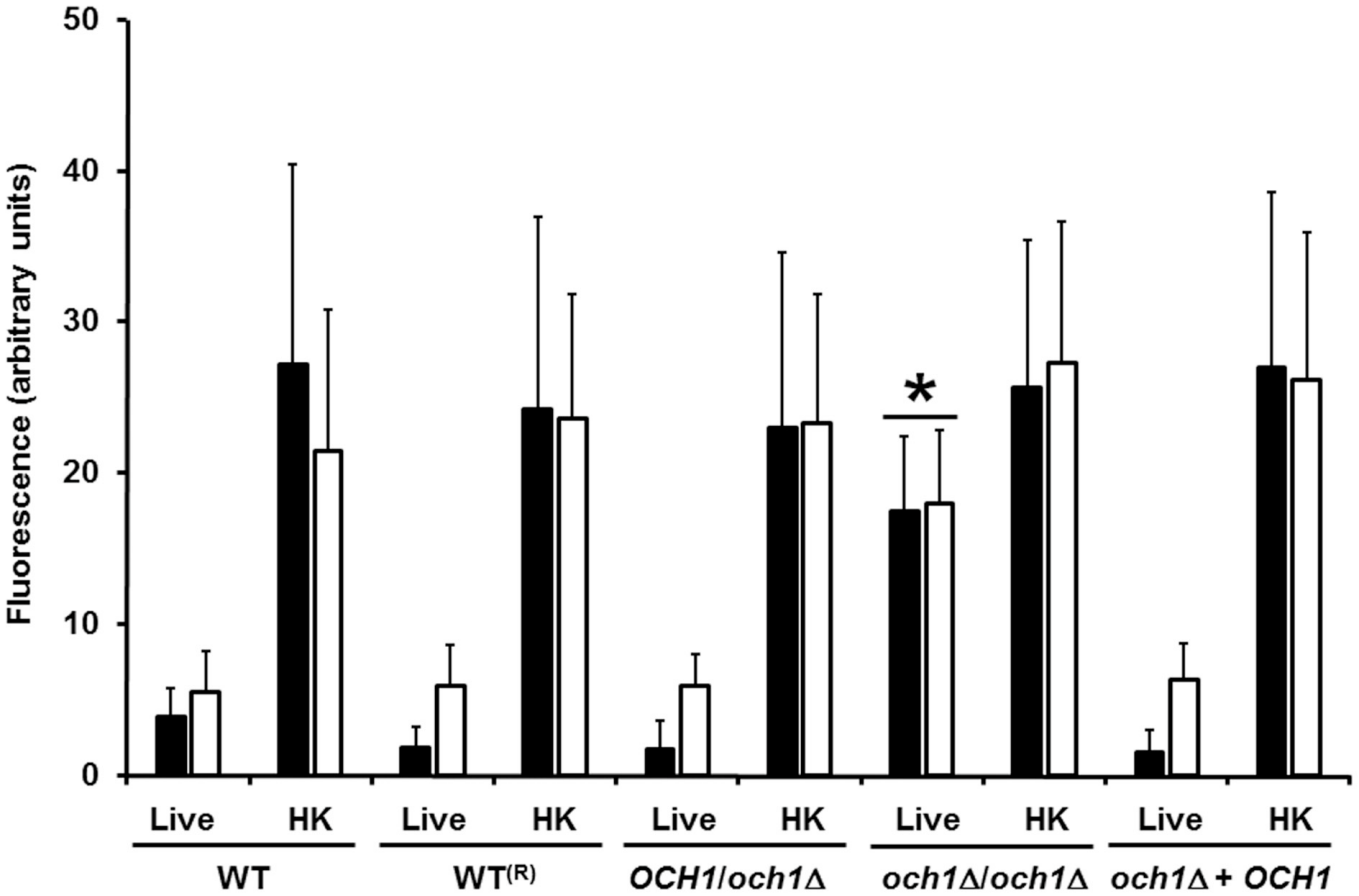
**Chitin and β1,3-glucan are significantly exposed at the cell surface of the *C. parapsilosis och1*Δ null mutant**. Live or heat-killed (HK) yeast cells were incubated with either fluorescein isothiocyanate-wheat germ agglutinin conjugate (closed bars) or IgG Fc-Dectin-1 chimera (open bars) as described in Materials and Methods, inspected under fluorescence microscopy, and the fluorescence associated to 50 individual cells recorded. The strains used are: CLIB-214 (WT), CPRI (WT^(*R*)^), AP (*OCH1*/*och1*Δ), AP-1 (*och1*Δ*/och1*Δ), and AP-2 (*och1*Δ + *OCH1*). ^*^P < 0.05, when compared with live cells from CLIB-214, CPR1, AP, and AP-2 strains.

We next assessed the cell wall composition of the Cp*och1*Δ null mutant. Cell walls were purified, acid hydrolyzed, washed and analyzed by High-Performance Anion-Exchange Chromatography with Pulsed Amperometric Detection (HPAEC-PAD). Results indicated similar carbohydrate content among the control strains, but the null mutant displayed a reduction of about 67% in the mannan content, along with a significant increment in β-glucan and chitin levels, when compared to the control strains (Table 1). No significant differences in the cell wall protein level or the phosphomannan content were observed (Table 1). The relative cell wall porosity to polycations, an indirect reporter of the glycan arrangement (De Nobel et al., 1990; Cheng et al., 2011), was greater in the Cp*och1*Δ null mutant than in the heterozygous or control strains (Table 1). Furthermore, we assessed whether rearrangements in the organization of cell wall components occurred upon disruption of Cp*OCH1*. For this, we quantified the ability of the fluorescein isothiocyanate-wheat germ agglutinin conjugate (WGA-FITC) and IgG Fc-Dectin-1 chimera to bind chitin and β1,3-glucan, respectively (Graham et al., 2006; Mora-Montes et al., 2011; Marakalala et al., 2013). Results shown in Figure 4 indicate that both lectins displayed a weak ability to bind either chitin or β1,3-glucan at the cell wall of live WT, reconstituted WT, heterozygous mutant and re-integrant control cells. However, the live *och1*Δ null mutant showed an increased ability to bind both WGA-FITC and the IgG Fc-Dectin-1 chimera (Figure 4). *C. albicans* inactivation by heat exposes inner cell wall components at the cell surface, such as β1,3-glucan and chitin (Gow et al., 2007; Mora-Montes et al., 2011). Thus, as expected, enhanced binding by both lectins occurred upon inactivation of yeast cells by heating (Figure 4). Altogether, these results indicate that the Cp*och1*Δ null mutant has significant defects in cell wall composition and fitness.

**Table 1:** **Cell wall analysis of Cp*och1*Δ null mutant and control strains**.

**Strain**	**Cell wall abundance**			**Phosphomannan content (μg)^a^**	**Porosity (%)^b^**	**Protein (μg)^c^**
	**Chitin (%)**	**Mannan (%)**	**Glucan (%)**			
CLIB-214 (WT)	2.27 ± 0.7	27.40 ± 0.7	70.32 ± 1.3	74.25 ± 3.3	65.32 ± 11.1	164.64 ± 20.6
CPRI (WT^R^)	2.15 ± 0.3	27.07 ± 4.6	70.76 ± 4.9	66.97 ± 8.3	71.52 ± 8.0	137.5 ± 46.3
AP (*OCH1/och1*Δ)	2.05 ± 0.5	26.13 ± 4.8	71.80 ± 4.7	66.95 ± 10.7	65.46 ± 8.9	148.17 ± 35.7
AP-1 (*och1*Δ*/och1*Δ)	4.94 ± 0.8^*^	9.26 ± 0.8^*^	85.78 ± 1.0^*^	68.03 ± 4.5	90.09 ± 3.8^*^	135.02 ± 17.4
AP-2 (*och1*Δ+ *OCH1*)	2.12 ± 0.5	26.97 ± 1.8	71.22 ± 1.8	70.77 ± 5.2	63.34 ± 7.9	155.40 ± 36.2
CLIB-214^d^ (WT)	2.27 ± 0.6	14.20 ± 1.3^*^	86.53 ± 1.8^*^	ND	ND	ND
AP-1^d^ (*och1*Δ*/och1*Δ)	4.24 ± 0.5^*^	Traces^*^	95.76 ± 0.5^*^	ND	ND	ND
AP-2^d^ (*och1*Δ + *OCH1*)	2.18 ± 0.4	14.64 ± 2.6	85.23 ± 1.1^*^	ND	ND	ND

a *μg of Alcian Blue bound/OD_600_ = 1*.

b *Relative to DEAE-Dextran*.

c *μg of protein/mg of cell wall*.

d *Upon β-elimination*.

*ND Not determined*.

* *P < 0.05*.

### Loss of *C. parapsilosis OCH1* affects cytokine production by human PBMCs

**Figure 5:**
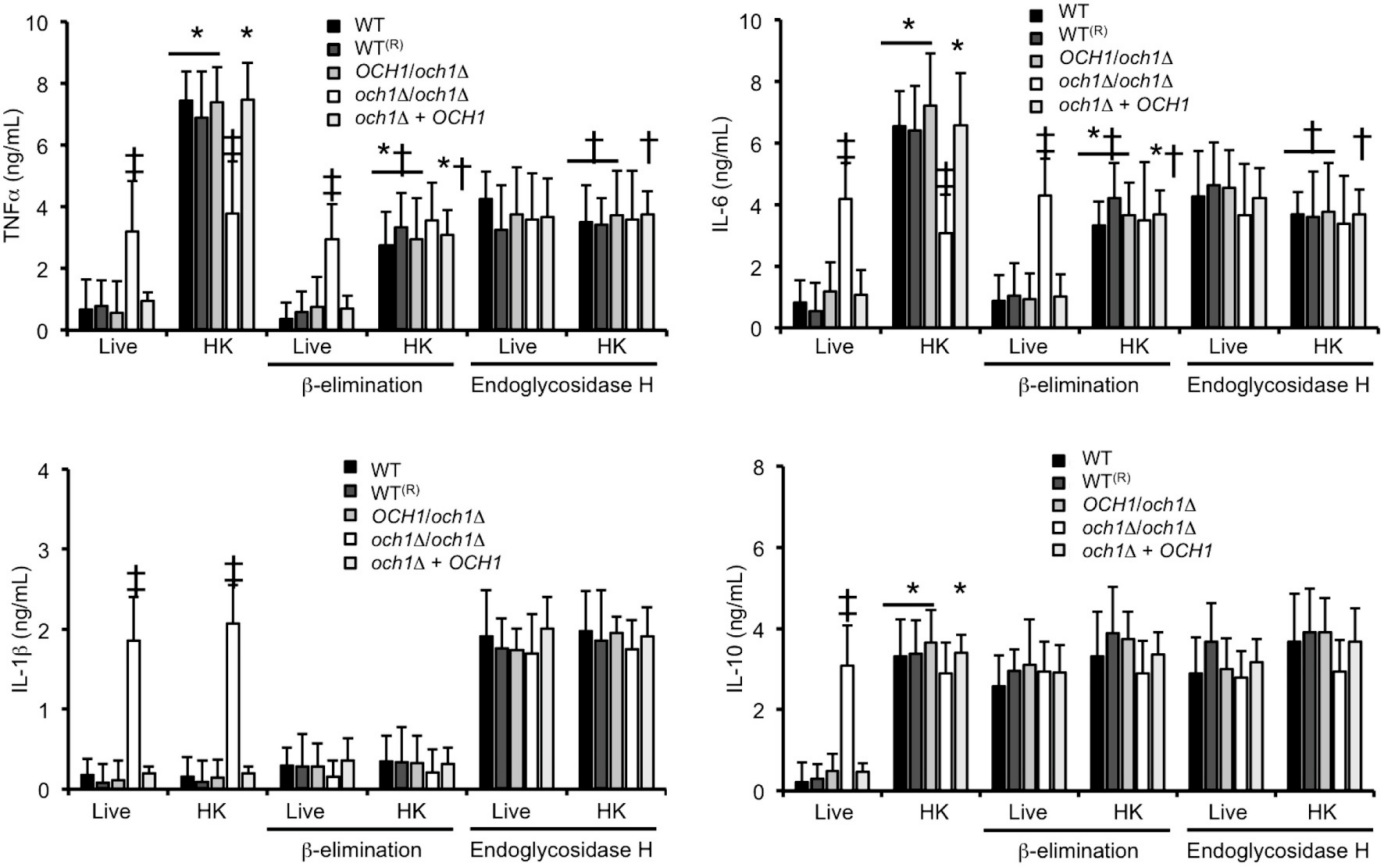
**Loss of proper cell wall mannosylation affects the ability of *C. parapsilosis* to stimulate cytokine production by human PBMCs**. Yeast cells where co-incubated with human PBMCs, the supernatant saved and used to quantify pro- and anti-inflammatory cytokines. Results (means ± SD) where obtained using samples from six donors, each assayed in duplicate wells. The strains used are: CLIB-214 (WT), CPRI (WT^(*R*)^), AP (*OCH1*/*och1*Δ), AP-1 (*och1*Δ*/och1*Δ), and AP-2 (*och1*Δ + *OCH1*). ^*^P < 0.05, when compared with live cells; ^†^*P* < 0.05, when compared with untreated cells; ^‡^*P* < 0.05, when compared with cells subjected to the same treatment.

We next assessed the relevance of proper cell wall *N*-linked mannosylation during *C. parapsilosis* interaction with human PBMCs, quantifying the levels of pro- and anti-inflammatory cytokines as a read out of this interaction. Live *C. parapsilosis* yeast cells from WT control strains and the heterozygous mutant stimulated low and similar TNFα, IL-1β, IL-6, and IL-10 levels (Figure 5). As mentioned, *C. albicans* inactivation by heat exposes β1,3-glucan at the cell surface, and heat-treated *C. albicans* cells are therefore more immunostimulatory compared to untreated cells (Gow et al., 2007). Here, upon heat inactivation of WT *C. parapsilosis* cells, the interaction with PBMCs resulted in stimulation of TNFα, IL-6, and IL-10, but not IL-1β (Figure 5). Live *C. parapsilosis och1*Δ null mutant stimulated significantly higher levels of both pro- and anti-inflammatory cytokines compared to WT and control cells (Figure 5), and, accordingly, WT and control cells treated with endo-H, i.e., with *N*-linked mannan enzymatically removed from the cell wall (Kobata, 1979), induced cytokine levels similar to that with the *och1*Δ null mutant cells (Figure 5). The heat-killed (HK) *och1*Δ null mutant and the endo-H-treated WT cells stimulated significantly less TNFα and IL-6 production than the heterozygous mutant, the reintegrant control and the WT control cells (Figure 5), suggesting that recognition of both *N*-linked mannans and inner cell wall components is required for maximal cytokine stimulation. Production of IL-1β and IL-10 was insensitive to heat killing in both the *och1*Δ null mutant and the endo-H-treated WT cells. Therefore, proper *N*-linked mannosylation is required for stimulation of cytokines by human PBMCs.

### Loss of *O*-linked mannans affects the ability of *C. parapsilosis* to stimulate cytokine production by human PBMCs

Next, to obtain insights into the role of *O*-linked mannans as PAMPs during *C. parapsilosis* sensing, we trimmed this oligosaccharide from the cell wall by β-elimination (Diaz-Jimenez et al., 2012), and used these cells to stimulate cytokine production by human PBMCs. After the removal of *O*-linked mannans, cell viability was not significantly altered (not shown), about 50% of mannan content was removed from the cell wall of WT and control cells, and no mannose was recorded in the cell wall preparations from *och1*Δ null mutant cells (Table 1). Furthermore, *C. albicans* β-eliminated cells stimulated similar cytokine levels than those stimulated by the *C. albicans mnt1-mnt2*Δ null mutant (Munro et al., 2005) that lacks long *O*-linked mannans from the cell wall (Figure 2S). Live WT control, heterozygous, re-integrant strain and *och1*Δ null mutant cells stimulated similar TNFα and IL-6 levels in comparison to the β-eliminated-treated cells (Figure 5). HK WT controls, re-integrant, and *OCH1/och1*Δ heterozygous cells, but not *och1*Δ null mutant cells, induced lower TNFα and IL-6 levels, suggesting that *O*-linked mannans significantly contribute to *C. parapsilosis* sensing by human PBMCs. Upon *O*-linked mannan trimming, HK *och1*Δ cells lost the ability to stimulate high IL-1β levels, suggesting a key role for this cell wall component in IL-1β stimulation. As in the case of *N*-linked mannans, loss of *O*-linked mannans did not affect production of IL-10 (Figure 5). Overall, these data indicate key roles of both *N*- and *O*-linked mannans during the interaction of *C. parapsilosis* with human PBMCs.

### Dectin-1 and TLR4 are required for IL-1β production

**Figure 6:**
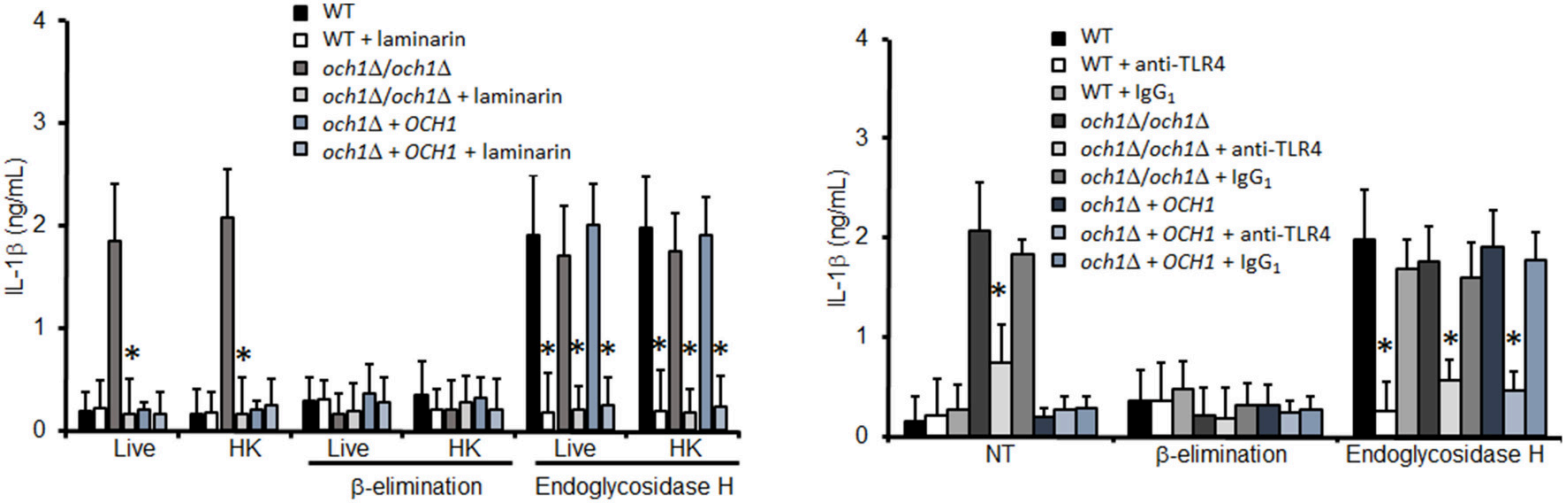
**Blocking of dectin-1 and TLR4 affects IL-1β stimulation by *C. parapsilosis* cells**. Human PBMCs were pre-incubated with either laminarin, antibody to TLR4, or irrelevant IgG_1_ for 1 h at 37°C, before incubation with yeast cells. After 24 h incubation at 37°C the supernatant were saved and used to quantify IL-1β. Results (means ± SD) where obtained using samples from six donors, each assayed in duplicate wells. The strains used are: CLIB-214 (WT), AP-1 (*och1*Δ*/och1*Δ), and AP-2 (*och1*Δ + *OCH1*). ^*^P < 0.05, when compared to same cell type without treatment. NT, non-treated cells.

Next, we analyzed the pathogen recognition receptors involved in the stimulation of IL-1β. The production of this cytokine was dependent on proper engagement of either dectin-1 or TLR4, as fungal cells lacking *N*-linked mannans stimulated reduced levels of IL-1β production upon blocking with laminarin and antibodies to TLR4, respectively (Figure 6). No effect was observed in the IL-1β levels when TLR2 was blocked with an antibody to TLR2, indicating this receptor does not affect stimulation of this cytokine (Figure 3S). As a control assay for this blocking agent, we found that this antibody did not affect the levels of IL-10 (Figure 4S), but significantly reduced TNFα production by WT cells harboring *N*-linked mannans at the cell wall (Figure 3S). Control experiments using isotype-matched, irrelevant antibodies did not show significant variations in cytokine stimulation by *C. parapsilosis* cells (Figure 6, Figure 3S). Overall, these data indicate that *N*-linked mannans are able to inhibit IL-1β production whereas stimulation of this cytokine requires presence of *O*-linked mannans, dectin-1 and TLR4.

### *N*-linked mannans are not required for the uptake of *C. parapsilosis* by macrophages

To assess the relevance of *C. parapsilosis* cell wall mannans during phagocytosis, primary human PBMC-derived macrophages were co-cultured with fluorescently labeled WT or *C. parapsilosis och1*Δ for 1.5 h and the ratio of phagocytosing macrophages was determined by flow cytometry. Interestingly, we found that *C. parapsilosis och1*Δ null mutant cells were as efficiently phagocytosed by the PBMC-derived macrophages as WT cells, indicating that *N*-linked mannans are not essential cell wall components for *C. parapsilosis* phagocytosis (Figure 5S). Furthermore, we similarly did not find a difference in the engulfment of *C. parapsilosis* WT or *och1*Δ null mutant cells by J774 mouse macrophages (data not shown).

### The *C. parapsilosis och1*Δ null mutant is attenuated in virulence in the mouse model of systemic candidiasis

**Figure 7:**
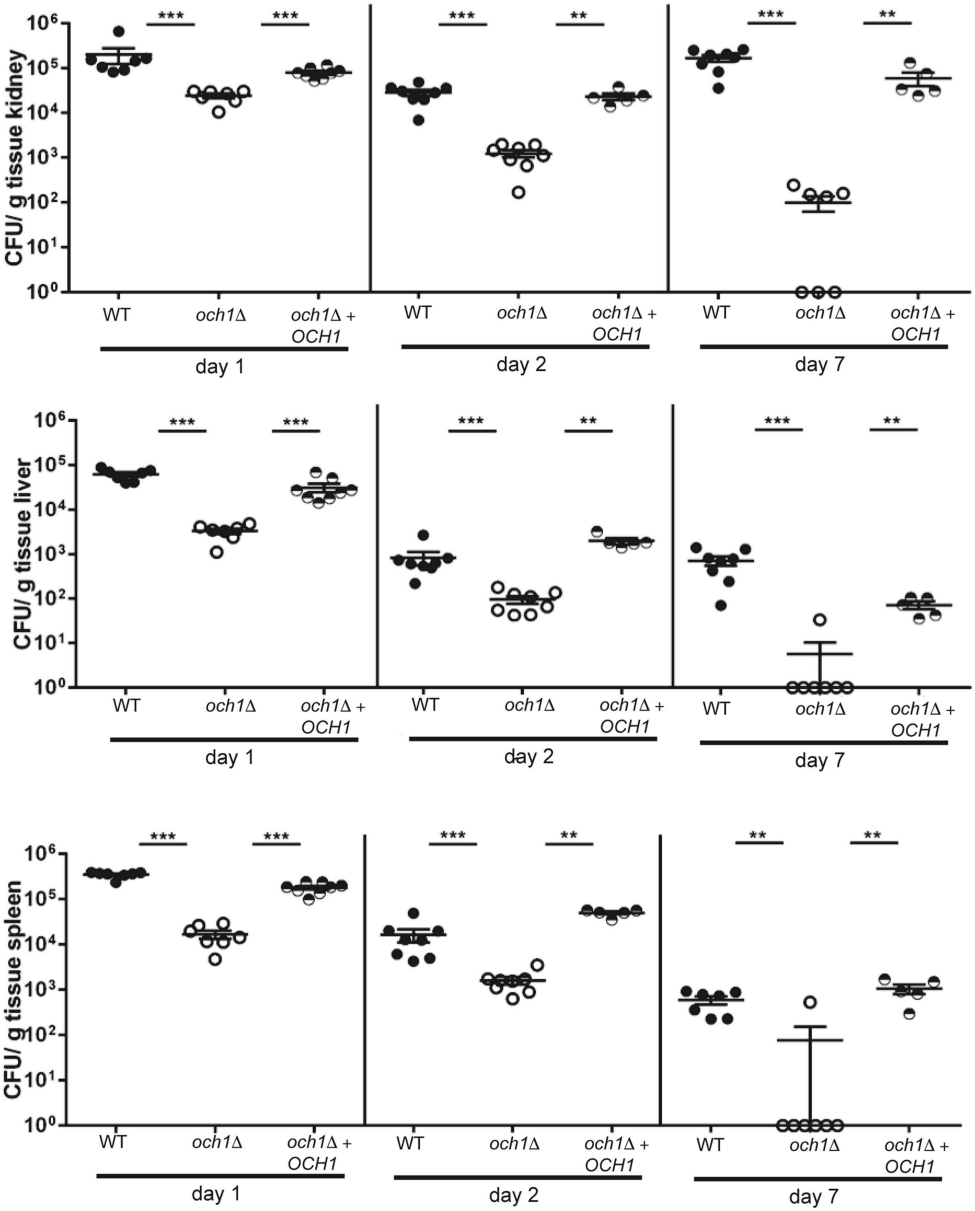
**The *C. parapsilosis och1*Δ null mutant has decreased virulence in the mouse model of systemic candidiasis**. Wild-type BALB/c mice were infected i.v. with 2 × 10^7^ cells from either *C. parapsilosis* wild type (CLIB-214), *och1*Δ (AP-1) or *och1*Δ + *OCH1* (AP-2), and fungal burdens in kidneys, liver, and spleen were determined at 1, 2, or 7 days post-infection and expressed as CFU/g tissue (mean ± SEM). Results are pooled data from 2 separate experiments with a total of 5–8 mice per group. ^**^P < 0.01, ^***^P < 0.001.

To determine how the disruption of the protein *N*-linked mannosylation affects the virulence of *C. parapsilosis*, we compared the susceptibility of BALB/c mice to *C. parapsilosis* WT and the *och1*Δ null mutant in a non-lethal experimental model of disseminated candidiasis, as previously reported (Ifrim et al., 2014). We found that mice infected with the *och1*Δ null mutant had significantly decreased fungal burdens in the spleen, kidneys and liver at 1, 2, and 7 days post-infection, when compared to those mice infected with the WT strain (Figure 7). Animals infected with the reintegrant control had fungal burdens similar to mice challenged with WT yeast cells (Figure 7). Therefore, these results demonstrate that the loss of *OCH1* significantly affects the virulence of *C. parapsilosis in vivo*.

We also assessed the virulence of the mutant strains generated in this work in the *Galleria mellonella* model of disseminated candidiasis (Gago et al., 2014; Jacobsen, 2014). Interestingly, larvae inoculated with either the WT control cells or the Cp*och1*Δ null mutant displayed similar mortality rates, with most of the larvae dying after 10 days post-inoculation (Figure 5S). As a control, we included the *C. albicans och1*Δ null mutant that was attenuated in a mouse model of systemic candidiasis (Bates et al., 2006), and this strain also displayed virulence attenuation in the *G. mellonella* infection system (Figure 6S). Therefore, these results indicate that the virulence of the *C. parapsilosis och1*Δ null mutant significantly depends on the host milieu.

## Discussion

Thus, far, there are a limited number of disrupted genes in *C. parapsilosis* and most of them are involved in either lipid metabolism or biofilm formation (Ding and Butler, 2007; Gácser et al., 2007; Ding et al., 2011; Nguyen et al., 2011a,b; Connolly et al., 2013; Holland et al., 2014). Here, we report the first disruption of a gene involved in *C. parapsilosis N*-linked mannosylation, and assess the contribution of this metabolic pathway in the fitness of this organism and its role in the interaction with the host. In addition, we also provide for first time a comparative analysis between enzymatic removal and gene disruption to evaluate the participation of *N*-linked mannans during the human PBMCs-fungus interplay.

Our studies indicate that the heterozygous strain at the *OCH1* locus, and the reintegrant control strain are functionally similar to the WT strain, indicating haplosufficiency, as reported in *C. albicans* (Bates et al., 2006). The *C. parapsilosis och1*Δ null mutant displayed typical phenotypes associated with defects in the *N*-linked mannosylation pathway, including slower growth rates, a clumpy cell phenotype, abnormal morphogenesis, and defects in the cell wall composition, porosity and fitness (Bates et al., 2005, 2006, 2013; Mora-Montes et al., 2007, 2010; Hall et al., 2013). Together with the complementation of the *C. albicans och1*Δ null mutant, these data strongly suggest Cp*OCH1* is the functional ortholog of *C. albicans OCH1.* Interestingly, the *C. parapsilosis* null mutant cells were not susceptible to hygromycin B, which strongly affects *Saccharomyces cerevisiae* and *C. albicans* cells with defects in the glycosylation pathways (Dean, 1995; Bates et al., 2005, 2006; Mora-Montes et al., 2007), suggesting that dependence on *N*-linked mannosylation for cell fitness is less important in *C. parapsilosis* compared to *C. albicans*. It has been reported that phosphomannosylation in *C. albicans* occurs in both, the *N*-linked mannan core and the outer chain, and the *OCH1* disruption significantly affected the cell ability to bind Alcian blue (Bates et al., 2006). Since the *C. parapsilosis* WT and *och1*Δ cells have a similar ability to bind Alcian blue, it is possible to suggest that phosphomannan is mainly attached to the glycan core or to the *O*-linked mannans, which contrast with the current knowledge about *C. albicans* mannan structure (Bates et al., 2006; Mora-Montes et al., 2007).

It is well established that cell wall mannans and β1,3-glucan play a pivotal role during the *C. albicans*-innate immune system interaction (Netea et al., 2008; Mora-Montes et al., 2009), and truncated *N*-linked mannans lead to a reduced ability to stimulate cytokine production by human PBMCs (Netea et al., 2006; Mora-Montes et al., 2007, 2010). Here, we found a similar result when HK *C. parapsilosis och1*Δ null mutant or endo-H-treated cells were used to stimulate TNFα and IL-6 production, indicating *C. parapsilosis N*-linked mannans do play a significant role during stimulation of these cytokines. However, in contrast to what has been reported in *C. albicans*, truncated *N*-linked mannans did not affect the stimulation of IL-10 production during HK *C. parapsilosis*-human PBMCs interaction, but the cytokine levels were diminished upon dectin-1 blocking with laminarin (data not shown). IL-10 stimulation can be triggered by engagement of dectin-1 with its ligand, and simultaneous stimulation of co-receptors, such as TLR2, amplifies the cytokine production (Reid et al., 2009). Thus, it is feasible to conclude that dectin-1 engagement by β1,3-glucan triggers the signaling pathway involved in IL-10 stimulation by *C. parapsilosis* cells. Results obtained here with live *C. parapsilosis och1*Δ cells, where IL-6, IL-10, and TNFα production was higher than in the WT control cells, could be explained by the reduction in the mannan levels and the increased β-glucan content at the cell wall surface of the null mutant. Therefore, it is likely that mannans mask the β1,3-glucan layer, and thus block the triggering of cytokine production via dectin-1, as reported (Gow et al., 2007; Wheeler and Fink, 2006).

The *C. albicans O*-linked mannans represent a minor component of the cell wall (Munro et al., 2005), and are a non-essential wall component for cytokine stimulation by human PBMCs, as loss of these oligosaccharides has little impact in the ability of *C. albicans* to stimulate production of both pro-and anti-inflammatory cytokines (Netea et al., 2006). Here, we found that in *C. parapsilosis* the *O*-linked mannans play a more significant role than that reported for *C. albicans*. Accordingly, we showed here that *O*-linked mannans represent about the half of the total mannan content of the *C. parapsilosis* wall, which might contribute to the differential role for this component in *C. albicans* and *C. parapsilosis*. The *C. parapsilosis* cells lacking both, proper *N*- and *O*-linked mannans displayed a reduction of about 50% in the stimulation of TNFα and IL-6, which contrast with the poor ability of *C. albicans pmr1*Δ null mutant (a strain with no mannans at the wall surface) to stimulate these cytokines (30% and 20% of TNFα and IL-6, respectively; Netea et al., 2006). Therefore, our results indicate that relevance of mannans during *C. parapsilosis* immune sensing is different from that described in *C. albicans*, and other *C. parapsilosis* wall components might play roles that are more important during interactions with immune cells.

**Figure 8:**
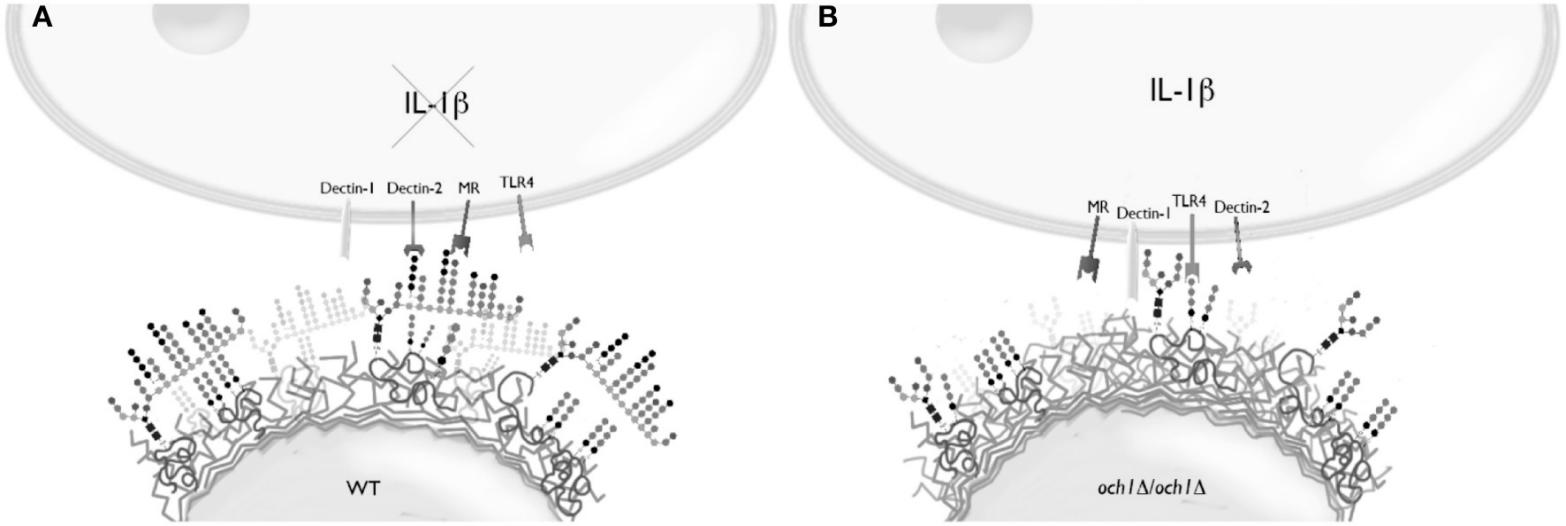
**Conceptualization of the stimulation of IL-1β production by *C. parapsilosis***. **(A)** In the wild-type cells, *N*-linked mannans mask both β1,3-glucan and the ligand of TLR4, most likely *O-*linked mannans, resulting in a relatively limited production of IL-1β. **(B)** Once the *N*-linked mannan outer chain is removed, such as in the *och1*Δ null mutant, the ligands for dectin-1 and TLR4 are exposed enabling the induction of a strong stimulation for IL-1β production.

Loss of proper cell wall *N*-linked mannosylation in *C. parapsilosis* led to an increased ability to stimulate IL-1β production and secretion, a product of inflammasome activation (van de Veerdonk et al., 2009), and this response was abrogated when *O*-linked mannans were removed from the cell wall, suggesting a key role for these oligosaccharides in IL-1β stimulation. Our data indicate the IL-1β production depends on engagement of β1,3-glucan with dectin-1 and TLR4 with its ligand, most likely *O*-linked mannans (Netea et al., 2006). Thus, our model to explain this results implies that *N*-linked mannans mask not only β1,3-glucans as in *C. albicans*, but also *O*-linked mannans (Figure 8). Hence, removal or disruption of the *N*-linked mannan layer results in the recognition of β1,3-glucan and *O*-linked mannans by dectin-1 and TLR4, respectively, leading to the stimulation IL-1β production. This model contrasts with the one reported for *C. albicans*, where *N*-linked mannans, dectin-1 and TLR2, but not *O*-linked mannans nor TLR4, are the key components in the signaling pathway for this cytokine stimulation (van de Veerdonk et al., 2009). These models therefore suggest differences in the structure of both *N*- and *O*-linked mannans in these fungal organisms. Accordingly, the *N*-linked mannans from *C. parapsilosis* are shorter and less complex than those found in *C. albicans* (Shibata et al., 1995). Notably, a cooperation between dectin-1 and TLR4 for IL-1β production has not been previously described.

The role of cell wall mannans during *C. albicans* phagocytosis by macrophages has been previously addressed and *N*-linked mannans are required for proper phagocytosis, while *O*-linked mannans play a negative role during this interaction (McKenzie et al., 2010). Interestingly, here we found that *N*-linked mannans are redundant wall components during *C. parapsilosis* phagocytosis by human and murine macrophages, suggesting once again a differential role for these cell wall glycans during host-pathogen interactions. Despite we did not find any significant difference in the uptake of both WT and *och1*Δ cells by phagocytic cells, it is not possible to discard a differential ability of macrophages to kill fungal cells, as it was not investigated in this work. When we assessed the relevance of *OCH1* during the host-fungus interaction, we found that *C. parapsilosis och1*Δ null mutant had significantly decreased virulence in a mouse model of systemic candidiasis, which is in line with the findings reported for *C. albicans* (Bates et al., 2006). However, it was interesting to observe that the *C. parapsilosis och1*Δ null mutant cells were as virulent as the control strain in the *G. mellonella* infection model, while the *C. albicans och1*Δ null mutant displayed virulence attenuation in both the insect and mouse models (Bates et al., 2006). Although, this insect model has been previously used to assess the virulence of members of the *C. parapsilosis* complex (Gago et al., 2014), our data suggest that this host might not be the most appropriate model to study the *C. parapsilosis* virulence. In addition, as phagocytic components play a very important role in the innate immune responses of *G. mellonella*, it is tempting to speculate that the decreased virulence of *C. albicans och1*Δ null mutant in this model results from its altered interaction with phagocytes.

In conclusion, we report that *OCH1* regulates *N*-linked mannan production in *C. parapsilosis* and impacts biological interactions with host effector cells. Deletion of *OCH1* leads to morphological alterations in *C. parapsilosis*, including cellular aggregation, inability to form pseudohyphae, and defects in the wall composition. Most significantly, *N*-linked and *O*-linked mannans differentially stimulate cytokine production during the interactions of *C. parapsilosis* with innate immune cells. Finally, the results further underscore the importance of detailed molecular and biological investigation of each of the members of the *Candida* genus.

## Experimental procedures

### Strains and culture conditions

The strains utilized in the experiments are listed in Table 2. Unless otherwise indicated, cells were maintained and propagated at 30°C in Sabouraud medium [1% (w/v) mycological peptone, 4% (w/v) glucose]. Two % (w/v) agar was included when solid medium was required. In order to prepare cells for cytokine assays and cell wall analyses, 500 μL from an overnight culture were transferred to 500 mL flasks containing 100 mL of fresh medium and incubated at 30°C with constant shaking at 200 rpm, until mid-log growth phase was reached. Filamentation was induced by growing 5 × 10^6^ cells/mL in RPMI 1640 medium (Sigma) supplemented with 10% fetal bovine serum (Sigma) or Lee's medium (all reagents from Sigma; Lee et al., 1975), at 37°C for 4 h. Cells were heat inactivated by incubating at 56°C for 60 min (Mora-Montes et al., 2007). Loss of cells viability was confirmed by absence of fungal growth in Sabouraud broth at 30°C for 72 h. β-Elimination was achieved by incubating cells overnight with 100 mM NaOH at room temperature and mild shaking (Diaz-Jimenez et al., 2012). Cells maintained viability upon β-elimination, since they showed no significant differences in CFU/mL before and after this treatment (average loss of cell viability upon β-elimination was 5.0 ± 2.0%). Trimming of *N*-linked glycans was accomplished by treating cells with endo-H (New England BioLabs) as described (Mora-Montes et al., 2012). The release of cell wall mannans was assessed by quantifying the free mannan content upon either β-elimination or treatment with endo-H, using HPAEC-PAD, in an ion chromatograph Dionex ICS-3000 (USA) with a guard-column CarboPac PA-100 (4 × 50 mm) and analytical CarboPac-PA100 (4 × 250 mm) column as reported (Mora-Montes et al., 2012). In some experiments, the medium was added with 2 units/mL chitinase (Sigma) to disperse cell aggregates as reported (Bates et al., 2006).

**Table 2:** **Fungal strains used in this work**.

**Strain**	**Organism**	**Origin**	**Genotype**	**References**
CLIB-214	*C. parapsilosis*	Clinical isolate	Type strain	Laffey and Butler, 2005
CPL2H1	*C. parapsilosis*	Derived from CLIB-214	*leu2*Δ*::FRT/leu2*Δ*::FRT, his1*Δ*::FRT/his1*Δ*::FRT*	Holland et al., 2014
CPRI	*C. parapsilosis*	Derived from CPL2H1	*leu2*Δ*::FRT/leu2*Δ*::FRT, his1*Δ*::FRT/his1*Δ*::FRT, FRT::*Cm*LEU2/FRT::*Cd*HIS1*	Holland et al., 2014
AP	*C. parapsilosis*	Derived from CPL2H1	*leu2*Δ*::FRT/leu2*Δ*::FRT, his1*Δ*::FRT/his1*Δ*::FRT, och1Δ::*Cm*LEU2/OCH1*	This work
AP-1	*C. parapsilosis*	Derived from AP	*leu2*Δ*::FRT/leu2*Δ*::FRT, his1*Δ*::FRT/his1*Δ*::FRT, och1Δ::*Cm*LEU2/ och1Δ::*Cd*HIS1*	This work
AP-2	*C. parapsilosis*	Derived from AP-1	As AP-1 but *RPSI*/*rps1*Δ::p*TDH3-OCH1-caSAT1*	This work
NGY152	*C. albicans*	Derived from CAI-4	*ura3*Δ-*iro1*Δ::*imm434*/ *ura3*Δ-*iro1*Δ::*imm434*; *RPSI*/*rps1*Δ::Clp10	Brand et al., 2004
NGY205	*C. albicans*	Derived from NGY204	*ura3*Δ-*iro1*Δ::*imm434*/ *ura3*Δ-*iro1*Δ::*imm434*; *och1*Δ::*hisG*/*och1*Δ::*hisG*	Bates et al., 2006
NGY357	*C. albicans*	Derived from NGY205	As NGY205, but *RPSI*/*rps1*Δ::Clp10	Bates et al., 2006
NGY328	*C. albicans*	Derived from NGY205	As NGY205, but *RPSI*/*rps1*Δ::Clp10-Ca*OCH1*	Bates et al., 2006
HMY163	*C. albicans*	Derived from NGY205	As NGY205, but *RPSI*/*rps1*Δ::Clp10-Cp*OCH1*	This work
NGY337	*C. albicans*	Derived from CAI-4	*ura3*Δ-*iro1*Δ::*imm434*/ *ura3*Δ-*iro1*Δ::*imm434*; *mnt1-mnt2*Δ::*hisG/mnt1-mnt2*Δ::*hisG, RPS1*/*rps1*Δ::CIp10	Munro et al., 2005
NGY335	*C. albicans*	Derived from NGY337	As NGY337 but *RPS1*/*rps1*Δ::CIp10-*MNT1*	Munro et al., 2005

### Construction of the Cp*och1*Δ null mutant, heterozygous and re-integrant strain

The gene disruption strategy performed here consisted in the use of a double auxotrophy (His^−^ Leu^−^) system in the CLIB-214 isolate of *C. parapsilosis* (CPL2H1 strain) (Holland et al., 2014), as described for *C. albicans* (Noble et al., 2010). Briefly, disruption cassettes containing either *C. dubliniensis HIS1* or *C. maltosa LEU2* genes flanked by ~500 nucleotides matching sequences upstream and downstream of the *C. parapsilosis OCH1* gene were generated by fusion PCR. The markers *C. dubliniensis HIS1* or *C. maltosa LEU2* were amplified by PCR from pSN52 or pSN40 (Noble and Johnson, 2005), respectively, using the same primers (primer pair: 5′-CCGCTGCTAGGCGCGCCGTGACCAGTGTGATGGA TATCTGC-3′and 5′-GCAGGGATGCGGCCGCTGACT CCGCTTAAACAATCGGCAAAGCTCGGATCCACTAGTAAC G-3′, underlined sequences align with the 5′ and 3′ *OCH1* fragments, respectively). The *C. parapsilosis OCH1* 5′ and 3′ regions of homology were amplified by PCR (primer pair: 5′-CAACATTTACATTCTTTTGC-3′ and 5′-CAC GGCGCGCCTAGCAGCGGATCAACGTATAAGCACTGC C-3′; and primer pair: 5′-GTCAGCGGCCGCATCCCTGCTGCC TGAAATGCTTACATAG-3′ and 5′-AACATCTCAAAACCGC AAGA-3′; homologous sequences to *C. dubliniensis HIS1* or *C. maltosa LEU2* markers are underlined, respectively). The heterozygous strain lacking one *OCH1* allele was constructed by chemical transformation of *C. parapsilosis* CPL2H1 with a *LEU2-*marked disruption cassette. Transformants were selected in minimal SC medium [0.67% (w/v) yeast nitrogen base, 2% (w/v) dextrose, 2% (w/v) agar] supplemented with L-histidine (1 mg/mL), and quick DNA extraction (Looke et al., 2011) was performed to screen Leu^+^ transformants by PCR, looking for the presence of expected 5′ and 3′ junctions of the integrated DNA. Homozygous *och1*Δ null mutant was constructed by transforming the heterozygous knockout strain with a *HIS1*-containing disruption cassette. Transformants were selected in minimal medium and quick DNA extractions (Looke et al., 2011) were performed to screen His^+^Leu^+^ mutants by PCR. Furthermore, to confirm the PCR results, genomic DNA samples were analyzed by means of southern blotting. Briefly, samples of genomic DNA from the null mutant, and control strains were digested with RI, run onto agarose gels, and transferred to nylon membranes. DNA samples were hybridized with specific probes targeting either *CmLEU2, CdHIS1*, or *CpOCH1* genes. These probes were amplified using Digoxigenin-marked dNTPs (Invitrogen) whose signal was revealed by enzymatic means (Roche). To reintegrate *OCH1* in the null mutant, the Invitrogen Gateway® cloning strategy was used. First the *OCH1* orf was amplified from the genomic DNA of *C. parapsilosis* CLIB 214. The 1089bp PCR product was purified using the PEG/MgCl_2_ method according to manufacturer's instruction from Invitrogen gateway cloning kit. Next, the BP clonase was used to integrate the *OCH1* ORF into the pDONR221 vector, and then subcloned into a modified destination vector, which contained a constitutive promoter, *pTDH3*, and a permanent *URA3* terminator sequence. Nourseothricin (NAT) was used as dominant selection marker. To achieve successful integration into the *C. parapsilosis RP10* locus, the destination vector was further modified by replacing the *C. albicans RP10* region to an artifical StuI restriction site containing *C. parapsilosis RP10* locus. The expression vector was then digested with StuI, and the linearized plasmid was transformed in the *och1*Δ*/och1*Δ null mutant by chemical transformation. Integration of the cassette in the *CpRP10* locus was checked by colony PCR and Southern blotting analysis. pDONR221 and Clp10-CaTDH3-GTW-URA3 plasmid vectors were kindly provided by Prof. Christophe d'Enfert.

### Heterologous complementation in *C. albicans*

To confirm that Cp*OCH1* is the functional ortholog of Ca*OCH1*, we performed the complementation of a Ca*och1*Δ null mutant (Bates et al., 2006). The Cp*OCH1* open reading frame, along with 1 Kbp upstream and 582 bp downstream sequences, was amplified by PCR (primer pair 5′- GCGGCCGCAGGCTAATCAAGAGGTTCCTG-3′ and 5′- GCGGCCGCTTCATAGCGAGTGAGAGAC-3′, with the bases to generate a NotI site underlined), cloned into pCR®2.1-TOPO® (Invitrogen), and subcloned into the NotI site of CIp10 (Murad et al., 2000). The resulting plasmid was linearized with StuI before transformation of the Ca*och1*Δ null mutant.

### Hex1 electrophoretic mobility shift assays

Cells in mid-log phase were collected by centrifugation, washed twice with deionized water, and mechanically broken, under a CO_2_ stream, in a Braun homogenizer during 5 min. Cell disruption was performed in cycles of 1 min, with 2-min resting periods on ice. Then, the homogenate was centrifuged for 10 min at 13 206 × g, 4°C and the supernatant was recovered. The samples were loaded onto a 4% PAGE gel and run for 11 h at 40 V under native conditions. The *N*-acetylhexosaminidase activity was determined by incubating with 0.4 mM 4-methylumbelliferyl N-acetyl-β-D-glucosamine (Sigma) in 0.1 M citrate-KOH buffer (pH 4.5) during 30 min at 37°C, and the results were observed by exposing the gel to UV light (Hernandez-Cervantes et al., 2012).

### Analysis of cell wall composition

Cells were mechanically broken as above described, the homogenate was centrifuged, the pellet recovered and extensively washed with deionized water for debris elimination. Cell walls were lyophilized and acid-hydrolyzed as described (Mora-Montes et al., 2007). Acid-hydrolyzed samples were analyzed by HPAEC-PAD and the carbohydrate separation was achieved with a gradient of sodium acetate in 150 mM NaOH (flow 0.5 mL/min) as follows: 0–5 min = 45–75 mM NaOH, 5.1–15.0 min = 90 mM NaOH, 15.1–17.0 min = 105 mM NaOH + 75 mM sodium acetate, 17.1–20.0 min = 75 mM NaOH + 150 mM sodium acetate, and 20.1–25.0 min = 45 mM NaOH, at a column temperature of 25°C. Applied potentials, for detection by the amperometric pulse were: E1 (400 ms), E2 (20 ms), E3 (20 ms), and E4 (60 ms) of + 0.1, −2.0, + 0.6, and −0.1 V, respectively. For quantification of cell wall protein content, lyophilized cell walls were alkali-hydrolyzed as reported (Mora-Montes et al., 2007), and analyzed using the Bradford protein assay.

### Cell wall porosity assay

Cell wall porosity was determined by relative porosity to polycations as described (De Nobel et al., 1990). Briefly, overnight-grown cells were inoculated into fresh Sabouraud broth, incubated for 4 h at 30°C and 200 rpm, and washed twice with PBS. Cell pellets containing 1 × 10^8^ cells were suspended in either 10 mM Tris-HCl, pH 7.4 (buffer A), buffer A plus 30 μg/mL poly-L-lysine (MW 30–70 kDa, Sigma Cat. No. P-2636) or buffer A plus 30 μg/mL DEAE-dextran (MW 500 kDa, Sigma Cat. No. D-9885), and incubated for 30 min at 30°C with constant shaking at 200 rpm. Preparations were centrifuged to pellet cells, supernatants recovered and further centrifuged before absorbance at 260 nm was measured. The relative cell wall porosity to DEAE-dextran was calculated as described (De Nobel et al., 1990).

### Alcian blue binding assays

Cells grown at mid-log phase were pelleted, washed twice with deionized water and adjusted at an OD_600_ of 0.2 in deionized water. Aliquots of 1 mL were pelleted and cells suspended in 1 mL of Alcian blue (Sigma; 30 μ g/mL, in 0.02 M HCl) and assayed as described (Hobson et al., 2004).

### Susceptibility to cell wall perturbing agents

Strains were tested for susceptibility to cell wall perturbing agents using a microdilution method as described (Bates et al., 2005). Briefly, cells from overnight culture in medium containing 2 units/mL chitinase were washed with water, passaged through a syringe with a 32-gauge needle, and suspended at an OD_600_ = 1. These cells were then inoculated into fresh medium at an OD_600_ of 0.01 and 95 μL of this suspension were dispensed into 96-well plates. A 5 μL volume of the cell wall perturbing agents was added to each well by duplicate across a range of double dilutions. The final OD_600_ was determined after 16 h of incubation at 30°C. The maximum concentrations tested for each agent were Calcofluor White (Sigma), Congo Red (Sigma) and tunicamycin (Sigma, 100 μ g/mL, each), SDS (BioRad, 0.1%, w/v), hygromycin B (Sigma, 500 μ g/mL), NaCl_2_ and KCl (1M, each), caffeine (Sigma; 50 mM) and vanadate (Sigma, 80 mM). Cells were grown in medium with no stressor added to normalize data. Growth data were normalized as percentage of those generated with the same strains without treatment.

### Fluorochrome staining

The assay was prepared as previously described (Mora-Montes et al., 2011). For chitin staining, cells were stained with 1 mg/mL WGA-FITC (Sigma). For β1,3-glucan staining cells were incubated with 5 μg/mL IgG Fc-Dectin-1 chimera (Graham et al., 2006) for 40 min at room temperature, followed by incubation with 1 μg/mL donkey anti Fc IgG-FITC for 40 min at room temperature (Marakalala et al., 2013). Samples were examined by fluorescence microscopy using a Zeiss Axioscope-40 microscope and an Axiocam MRc camera. From the pictures acquired, the fluorescence quantification of 50 cells was achieved using Adobe Photoshop™ CS6 with the next formula: [(total of green pixels-background green pixels) × 100]/total pixels.

### Ethics statement

The Ethics Committee from Universidad de Guanajuato approved the use of human cells in this study (permission number 17082011); while University of Szeged granted permission XII./00455/2011 to work with mice. Human cells were collected from healthy adult volunteers after information about the study was provided and written informed consent was obtained.

### Isolation and stimulation of human PBMCs with *Candida* cells

Human PBMCs were isolated by density centrifugation using Histopaque-1077 (Sigma) as described (Endres et al., 1988). For stimulation experiments, the interactions were performed in round-bottom 96-well microplates with 100 μL of cells adjusted to 5 × 10^5^ PBMCs in RPMI 1640 Dutch modification (added with 2 mM glutamine, 0.1 mM pyruvate and 0.05 mg/mL gentamycin; all reagents from Sigma) and 100 μL with 1 × 10^5^ fungal cells freshly harvested or treated. The interactions were incubated for 24 h at 37°C with 5% (v/v) CO_2_. In some experiments, PBMCs were preincubated for 1 h at 37°C with either laminarin (200 μg/mL), anti-TLR2 (10 μg/mL, eBioscience, Cat. No. 16-9922) or antibodies to TLR4 (10 μg/mL, Santa Cruz Biotechnology, Cat. No. sc-293072) prior to stimulation with *Candida* cells. Isotype matched, irrelevant antibodies, IgG_2_aκ (10 μg/mL, eBioscience, Cat. No. 14-4724-85) and IgG_1_(10 μg/mL, Santa Cruz Biotechnology, Cat. No. sc-52003) were used as controls for experiments assessing TLR2 and TLR4, respectively. All reagents used for the pre-incubation experiments were negative to contamination with LPS (tested with the *Limulus* amebocyte lysate from Sigma), and all reactions were performed in presence of 5 μ g/mL polymyxin B (Sigma) (Schwartz et al., 1972). Plates were centrifuged for 10 min at 3000 × g at 4°C, the supernatant saved and kept at −20°C until used.

### Cytokine quantification

The concentration of TNFα, IL-6, and IL-10 was quantified by ELISA (Peprotech), according to the manufacturer's instructions. The IL-1β levels were measured using a commercial ELISA kit from R&D Systems.

### Differentiation of human PBMC-derived macrophages

After isolation, aliquots of 1 mL containing 2 × 10^7^ PBMCs in RPMI supplemented with 1% (v/v) penicillin-streptomycin solution (PS, Sigma-Aldrich) were placed in flat bottom 12-well plates, and incubated 1.5 h at 37°C, 5% (v/v) CO_2_. Non-adherent cells were removed gently with the medium and adherent cells were washed with PBS at 37°C. One mL of X-VIVO 15 serum-free medium (Lonza) supplemented with 1% (v/v) PS and 10 ng/mL recombinant human granulocyte-macrophage colony-stimulating factor (GM-CSF, Sigma) were added into each well and incubated for 6–7 days at 37°C, 5% (v/v) CO_2_, with fresh medium exchanged every 2–3 days (Netea et al., 2009).

### Phagocytosis assays

Phagocytosis of *C. parapsilsosis* cells was performed as previously described (Nemeth et al., 2013). Briefly, *C. parapsilosis* cells were labeled with AlexaFlour488 Succinimidyl ester (Life Technologies) and subsequently co-incubated with human PBMC-derived macrophages at an effector:target ratio of 1:5 for 1.5 h. Non-phagocytosed yeast cells were removed by gentle washing and macrophages were detached from cell culture plates by using TrypLE™ Express solution (Gibco). To inhibit phagocytosis, macrophages were pre-incubated for 30 min with 2.5 μM cytochalasin D (R&D Systems) before infection. Samples were measured on a FACSCalibur instrument and analyzed by FlowJo vX.0.7 software.

### *C. parapsilosis* infection model and fungal burden

A non-lethal experimental model of disseminated candidiasis was used as reported previously (Ifrim et al., 2014). Briefly, groups containing 8–12-weeks-old male Balb/c WT mice (22–27 g. of weight) were injected via the lateral tail vein with 2 × 10^7^ *C. parapsilosis* cells, previously passaged through a syringe with a 32-gauge needle, in 100 μL of sterile PBS. A group of control mice was injected with 100 μL of sterile PBS. Animals were mantained with sterile water and pet aliment *ad libitum*. After 1, 2, or 7 days post infection, animals were humanitarianly euthanatized, and liver, kidneys and spleen were aseptically removed, weighed, and homogenized in sterile PBS in a tissue grinder. The fungal burden in these tissues was determined by plating serial dilutions on three YPD agar plates per tissue. The CFU were counted after 48 h of incubation at 30°C and expressed as CFU/g tissue.

### *G. mellonella* survival assays

Wax moth larvae killing assays were performed as described (Mylonakis et al., 2005). Briefly, 10 μL of a cell suspension prepared in PBS and containing 2 × 10^7^ yeast cells, passaged through a syringe with a 32-gauge needle, were injected directly into the haemocele, through last left pro-leg of the larva, using a 26-gauge needle and a Hamilton syringe. Larvae were incubated at 25°C after injection and survival monitored daily. Groups of 10 larvae were used for each strain analyzed. A group of untreated larvae and a PBS-injected group were included in each experiment as controls.

### Statistical analysis

Statistical analysis was performed using GraphPad Prism 6 software. Results obtained upon incubation with the cell wall perturbing agents were analyzed by two-way ANOVA. Cytokine stimulation using human PMBCs was performed in duplicate with six healthy donors, whereas the rest of the experiments were performed at least thrice in duplicate. Data represent cumulative results of all experiments performed. The Mann-Whitney *U* test or unpaired *t*-test was used to establish statistical significance (see figure legends for details), with a significance level set at *P* < 0.05.

## Author contributions

AF, AG, and HM conceived the study. LP, KC, EE, EM, TN, LL, RT, CV, AM, and AT performed experiments. LP, AF, ML, JN, AG, and HM analyzed data. LP, JN, AG, and HM drafted the manuscript. LP, KC, AF, EE, EM, TN, LL, RT, ML, CV, AM, AT, JN, AG, and HM revised and approved the manuscript. AG and HM equally contributed to this work, and are corresponding authors.

### Conflict of interest statement

The authors declare that the research was conducted in the absence of any commercial or financial relationships that could be construed as a potential conflict of interest.
