# Supplementary material for: Role of Protein Glycosylation in Candida parapsilosis Cell Wall Integrity and Host Interaction
Source: Front Microbiol. 2016 Mar 8;7:306. doi: 10.3389/fmicb.2016.00306 (PMC4781877; doi:10.3389/fmicb.2016.00306)
Supplement: Supplementary file 1 [file DataSheet1.docx]

Supplementary Material

**Role of Protein Glycosylation in *Candida parapsilosis* Cell Wall Integrity and Host Interaction**

**Luis A. Pérez-García^1^, Katalin Csonka^2^, Arturo Flores-Carreón^1^, Eine Estrada-Mata^1^, Erika Mellado-Mojica^3^, Tibor Németh^2^, Luz A. López-Ramírez^1^, Renata Toth^2^, Mercedes G. López^3^, Csaba Vizler^4^, Annamaria Marton^4^, Adél Tóth^2^, Joshua D. Nosanchuk^5^, Attila Gácser^2,^*, and Héctor M. Mora-Montes^1,^*.**

^1^Departamento de Biología, División de Ciencias Naturales y Exactas, Campus Guanajuato, Universidad de Guanajuato, Noria Alta s/n, col. Noria Alta, C.P. 36050, Guanajuato, Gto., México

^2^Department of Microbiology, University of Szeged, Közép fasor 52, H-6726 Szeged, Hungary

^3^Centro de Investigaciones y de Estudios Avanzados del IPN, Apartado Postal 629, 36500 Irapuato, Guanajuato, México,

^4^Biological Research Centre, Hungarian Academy of Sciences, H-6726 Szeged, Hungary

^5^Albert Einstein College of Medicine, Jack and Pearl Resnick Campus, Bronx, NY 10461, U.S.A.

*** Correspondence:** Both authors equally contributed to this work: Attila Gacser, Tel: +36/62544849, Fax: +36/62544823 Email: gacsera@gmail.com; Hector M. Mora-Montes, Tel. (+52) 473-7320006 Ext. 8154; Fax (+52) 473-7320006 Ext. 8153; e-mail: hmora@ugto.mx

# Supplementary Data





**Figure 1S: Generation of the *C. parapsilosis och1*Δ mutant**. (A) The *CpOCH1* was disrupted using a double auxotrophy (His- Leu-) system derived from *C. parapsilosis* CLIB-214. Disruption cassettes containing either *C. dubliniensis HIS1* or *C. maltosa LEU2* genes replaced each *OCH1* allele in sequential rounds of transformation, restoring the phenotypes His^+^ and Leu^+^. The Cm*LEU21* and Cd*HIS* disruption cassettes were flanked by ~500 nucleotides matching sequences upstream and downstream of the *C. parapsilosis OCH1* gene were generated by fusion PCR. (B) Generation of the null mutant was confirmed by southern blotting using a specific probe targeting the Cp*OCH1*gene. Probes targeting Cm*LEU2* or Cd*HIS1* genes were used to discard ectopic integrations. The strains used are: CLIB-214 (WT), CPRI (WT^(R)^), AP (*OCH1*/*och1*Δ), and AP-1 (*och1*Δ*/och1*Δ).





**Figure 2S: Cytokine stimulation by *C. albicans* cells upon β-elimination.** Yeast cells where co-incubated with human PBMCs, the supernatant saved and used to quantify pro-inflammatory cytokines. Bars represent means ± SD using samples from six donors each assayed in duplicates. The strains used are: NGY152 (WT), NGY337 (*mnt1*Δ-*mnt2*Δ), and NGY335 (*mnt1*Δ-*mnt2*Δ + *MNT1*).


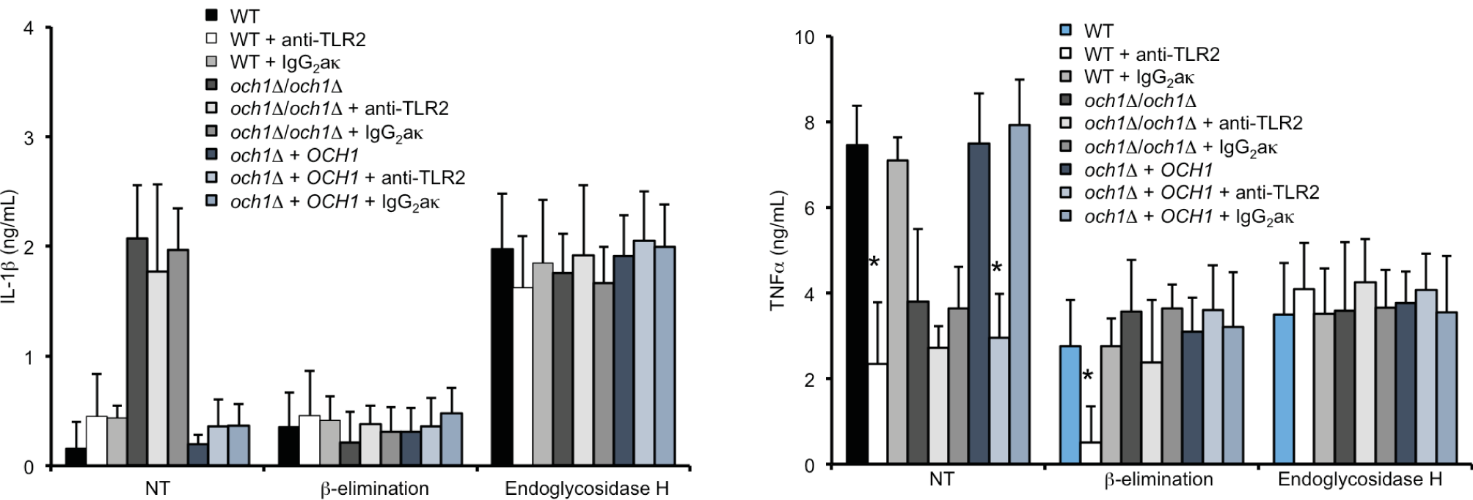


**Figure 3S: Blocking of TLR2 affects TNFα production, but not IL-1β stimulation, by *C. parapsilosis* cells.** Human PBMCs were pre-incubated with either antibody to TLR2, or irrelevant IgG_2ak_ for 1h at 37°C, before incubation with HK yeast cells. After 24 h incubation at 37°C the supernatant were saved and used to quantify IL-1β and TNFα. Results (means±SD) where obtained using samples from six donors assayed in duplicates. The strains used are: CLIB-214 (WT), CPRI (WT^(R)^), AP (*OCH1*/*och1*Δ), AP-1 (*och1*Δ*/och1*Δ), and AP-2 (*och1*Δ *+ OCH1*). **P* < 0.05, when compared to same cell type without treatment. NT, Non-treated cells.


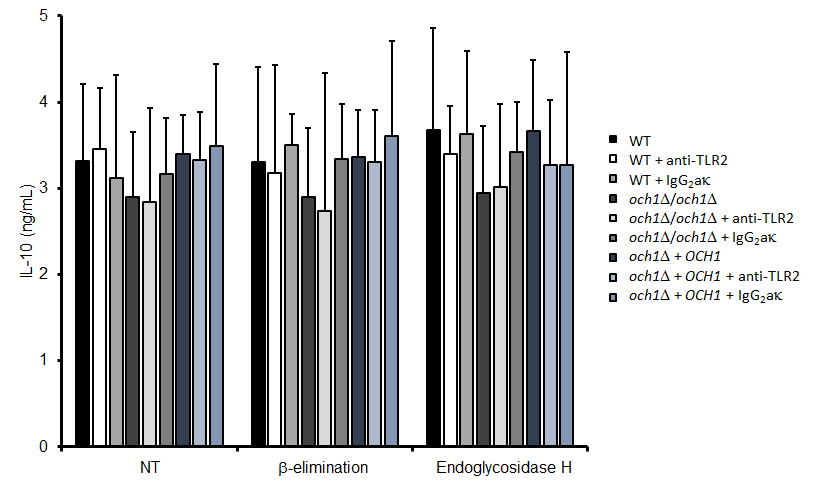


**Figure 4S. Blocking of TLR2 does not affect IL-10 stimulation by *C. parapsilosis* cells.** Human PBMCs were pre-incubated with either antibody to TLR2, or irrelevant IgG_2ak_ for 1h at 37°C, before incubation with HK yeast cells. After 24 h incubation at 37°C the supernatant were saved and used to quantify IL-10. Results (means±SD) where obtained using samples from six donors assayed in duplicates. The strains used are: CLIB-214 (WT), CPRI (WT^(R)^), AP (*OCH1*/*och1*Δ), AP-1 (*och1*Δ*/och1*Δ), and AP-2 (*och1*Δ *+ OCH1*). NT, Non-treated cells.


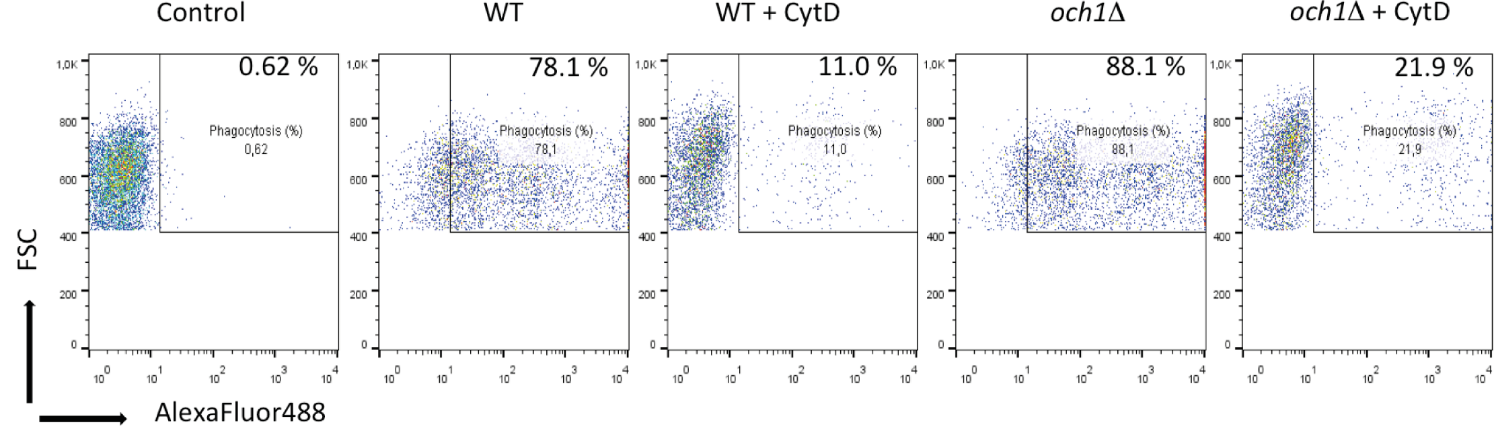


**Figure 5S: The lack of *OCH1* does not affect the *C. parapsilosis* phagocytosis by primary human macrophages.** Primary human PBMC-derived macrophages were co-incubated with AlexaFluor488-labeled *C. parapsilosis* cells (MOI 5) for 1.5 h and the ratio of macrophages associated with yeast cells was determined by flow cytometry. To exclude adhesion events, association was also examined in the presence of the phagocytosis inhibitor cytochalasin D (CytD). Results are representative of three independent experiments.





**Figure 6S. The *C. parapsilosis och1*Δ null mutant does not display virulence attenuation in the *G. mellonella* model.** Aliquots containing 2x10^7^ yeast cells were injected directly into the haemocele of *G. mellonella* larvae, and survival was monitored daily. Groups of ten larvae were used for each strain analyzed. PBS, PBS-injected group included as control. The strains used are: CLIB-214 (Cp WT), AP-1 (Cp *och1*Δ), NGY152 (Ca WT), NGY357 (Ca *och1*Δ), and NGY328 (Ca *och1*Δ + *OCH1*).
